# Biographical Anecdotes
*Compiled chiefly from the accounts published by Dr. Hird, Dr. Elliot, Dr. Thompson, and Dr. Lettsom; —we mention them in the order in which they have appeared.


**Published:** 1783

**Authors:** 


III. *Biographical Anecdotes* of the late* John Fothergill, M. D. F. R. S. and S. A. *Member of the Royal College of Phyſicians at London and Edinburgh; of the Royal Medical Society at Paris; and of the American Philoſophical Society.*

THIS celebrated phyſician was born on the 8th of March 1712, at Carr-end, in the county of York. His parents were John † and Margaret Fothergill, both perſons of approved integrity, whoſe names are ſtill dear to the ſociety of Quakers. He was one of many children, but not the only one who excelled in the gifts of underſtanding.

* Compiled chiefly from the accounts publiſhed by Dr. Hird, Dr. Elliot, Dr. Thompſon, and Dr. Lettſom; —we mention them in the order in which they have appeared.

† "In the year 1734, one John Fothergill, pro-"bably the Doctor's father, and Joſeph Stor, took a "very active part in the conteſted election for the "county of York, and ſigned a circular letter to the "Quakers, recommending to their favour Sir Rowland "Wynn and Cholmondeley Turner, two of the candi-"dates."——Dr. Elliot's Life of Dr. F.

He

He was early in life under the care of his grandfather, Mr. Thomas Hough, a perfon of fortune, who refided at Frodfham in Chefhire, and who at a proper age placed him at the grammar fchool in that town. At this fchool he continued till his twelfth year, and was then removed to another at Sedberg in Yorkfhire, where he remained about four years, and attained to a competent knowledge of the Latin tongue, and fome acquaintance with the Greek; and though, as he himfelf has been heard to acknowledge*, he was not careful enough to improve the latter, it is certain that after he left thefe fchools he continued to cultivate his Latinity, fo as to read authors in that language very familiarly, and to write in it with fufficient fluency. But his attention was directed rather to general knowledge than to the ftudy of the learned languages, which he regarded little farther than as the vehicles of profitable information.

About the year 1728, he was put apprentice to Benjamin Bartlett, a very reputable and intelligent quaker apothecary, at Bradford in Yorkfhire, who before this had been the tutor of Dr. Hillary.

After having completed the term of his apprenticefhip with fidelity and reputation, he removed to Edinburgh, to ftudy phyfic under the doctors Monro, Alfton, Rutherford, Sinclair, and Plummer, who at that time filled the profefforial chairs in that univerfity with diftinguifhed re-

* Dr. Thompfon's Life of Dr. F.

putation. Here he remained till the year 1736, diligently employed in his profeffional purfuits, and conciliating the particular regard of the profeffors.

During his refidence at the Univerfity he formed a friendfhip which continued through life, with three of his fellow ftudents of diftinguifhed abilities. Thefe were Dr. Ruffell, who wrote the hiftory of Aleppo, and whom he has commemorated in a very elegant eulogium †; Dr. Cleghorn, of Dublin, author of an excellent work on the difeafes of Minorca; and Dr. Cuming, of Dorchefter.

It was on the 13th of Auguft 1736, that he graduated at Edinburgh. On this occafion he defended a thefis "Re emeticorum ufu," which has been thought worthy of being preferved in Smellie's Thefaurus. On the 29th of the following month he arrived in London, and immediately entered himfelf phyficians pupil at St. Thomas's hofpital, the practice of which he diligently attended for two years.

In the fpring of the year 1740, he was folicited to accompany a few friends in a vifit to the Continent. They were perfons of too many engagements at home to admit of long refidence in any one fpot, but a journal of the tour kept by the doctor, and communicated, on his return, to his friend Dr. Cuming, in a Latin letter, indicates a genius turned for general obfervation

† An Effay on the character of the late Alexander Ruffell, M. D. F. R. S. read before the fociety of phyficians, the 2d of October 1769.

and and curiofity. From this letter it appears, that after paffing through Flanders to Bruffells, the doctor travelled on to Spa and Aix la Chapelle, and from thence through Holland and Friefland to Bremen.

After this excurfion to the Continent doctor Fothergill returned to London, and took up his refidence in White-hart Court, Grace-church Street, where he continued during the greater part of his life, and where he acquired and eftablifhed both his fame and fortune. His bufinefs for fome years was confined chiefly to the lower clafs of people, fo that he has often traverfed † the outfkirts of the city from morning till night, and returned home without having taken one fee.

In 1746, he was admitted a licentiate of the college of phyficians, and two years after this, viz. in 1748, his "account of the putrid fore throat made its appearance." To this piece, which is entitled to great praife, and has been tranflated into almoft every European language, he owed a confiderable extenfion of his practice. He was now introduced into the firft families in the metropolis; and he was rarely ever employed, but in emergencies he was fought for again. His popularity has by fome been afcribed to the novelty of his character; but the fact is, that at that time there were two other phyficians in London of the fame religious perfuafion as himfelf. A peculiar addrefs and fuperior talents

† Dr. Thompfon's Life of Dr. F.

were no doubt the caufes to which he owed his fuperiority.

In April 1750, he began to publifh in the Gentleman's Magazine a monthly account of the weather and difeafes in London, which was regularly continued for feveral years. But in 1755, we find him "in doubt whether he fhall be able "to furnifh even fo much as he had done, for "the future; being partly prevented by want of "health and proper leifure, and partly difcou-"raged by an apprehenfion that what he did "was of fo little confequence as to be difre-"garded †." The doctor meant to excite other experienced phyficians in different parts to imitate his example, by communicating fimilar obfervations; but being difappointed in his views, and his time being much engroffed in practice, he difcontinued the publication in the beginning of the year 1756.

In 1754 he was elected a fellow ‡ of the college of phyficians at Edinburgh, and in 1763 a fimilar honour was conferred on him by the Royal Society of London, to whom he had feveral years before this communicated fome interefting papers, inferted in different volumes of their tranfactions. Thefe were not the only academical honours which his great merit procured him. He was one of the earlieft members of the American Philofophical Society, and in 1776, when a Royal Medical Society was inftituted

† Gent. Mag. Jan. 1755.

‡ An *ordinary*, not an *honorary*, fellow, as mentioned by Dr. Thompfon.

at Paris, Dr. Fothergill was one of a ſelect number of foreign phyſicians whom the ſociety thought proper to rank amongſt their aſſociates *.

In this enumeration of the compliments paid to his zeal and abilities we muſt not omit to mention that the celebrated Linnæus diſtinguiſhed a ſpecies of *Polyandria Digynia*, by the name of *Fothergilla Gardeni*.

In the conteſt between the fellows and licentiates of the College of Phyſicians, Dr. Fothergill took an active part, and ſubſcribed five hundred pounds towards bringing it to a legal deciſion. When all thoughts of law proceedings were laid aſide, the licentiates continued to aſſemble once a month for the ſake of reading medical papers, and converſing on the prevailing diſeaſes. After the death of Sir William Duncan, Bart. Dr. Fothergill was unanimouſly elected to be their preſident, and he continued in this office till his death. He was ſeldom abſent from their meetings, and his uniform endeavours to render this inſtitution a ſource of profeſſional harmony, and at the ſame time of public utility, raiſed him high in the eſteem of every member.

* The members of this ſociety are divided into two claſſes, viz. Aſſociates or Fellows (*Socii*) and Correſpondents (*Correſpondentes*); the number of the former is limited to 60, whereas that of the latter is unlimited. Dr. Lettſom, through miſtake, ſtiles Dr. F. a correſponding member.

Ever

Ever anxious to promote the interefts of his profeffion by extending our knowledge of difeafes, he united himfelf with a felect number of ingenious phyficians, in collecting materials for a work of which five volumes have appeared under the title of *Medical Obfervations and Enquiries*, a performance known and efteemed in every country where medical fcience is fuccefsfully cultivated. Befides the numerous effays in this excellent collection to which the name of Dr. Fothergill is prefixed, we now learn † that he was likewife the author of the three anonymous papers in the fourth volume.

In 1774, when the legiflature became juftly alarmed at repeated inftances of infection, which prifoners diffeminated in courts when brought before their judges, Dr. Fothergill and his humane friend Mr. Howard, were defired to attend the houfe of commons; before which they gave fuch information as induced the parliament to pafs a bill for preventing the gaol diftemper, and afterwards to recommend the building of penitentiary houfes, as a mode of punifhment calculated to reftrain indolence and vice. Thefe two diftinguifhed perfons, with George Whatley, efq. were appointed commiffioners for carrying into execution this new fyftem of correction.

A man of his diftinguifhed abilities and mild difpofition could not fail to acquire the efteem of his peaceful brethren, the quakers; and accordingly he was for many years looked up to as one of their moft valuable members, and was frequently

† Dr. Lettfom's account of Dr. F.

appointed to draw up and ſign the annual letter to the ſociety at their general aſſembly at Whitſuntide. He likewiſe compoſed and preſented the congratulatory addreſs of the quakers to his preſent majeſty on his acceſſion to the throne, which he managed very ingeniouſly, avoiding the peculiarities of ſpeech to which thoſe of his ſect are ſo much addicted.

To paſs through life perfectly free from cenſure is the lot of few. Perhaps no man ever deſerved it leſs than Dr. Fothergill; but an unfortunate diſpute with a perſon of his own religious perſuaſion expoſed him to the ill-natured reflections of many of his own ſect as well as of the public at large. It clearly appeared, however, that this obloquy was perfectly unmerited on the ſide of Dr. Fothergill. The circumſtances alluded to were as follows: in the year 1766, one Samuel Leeds, an illiterate perſon, who had been brought up to the trade of a bruſh-maker, was admitted by the univerſity of Edinburgh to the degree of doctor of phyſic; and on his coming to London ſoon afterwards, being eſpouſed by ſeveral leading perſons amongſt the quakers, was choſen phyſician to the London hoſpital. Soon after his election, one of his colleagues, in a converſation with Dr. Fothergill, happening to mention Leeds's ſucceſs, the doctor replied, "Take care he does no miſchief." It was not long before Leeds gave ſufficient marks of his ignorance to alarm the governors; and what had dropped from ſo reſpectable a perſon as Dr. Fothergill might perhaps not a little tend to convince them of their precipitancy in electing ing a phyſician to their hoſpital ſo unqualified for the duties of it. They therefore made a reſolution "that no phyſician ſhould continue to "officiate in that hoſpital, who had not paſſed "an examination at the college of phyſicians." Dr. Leeds now ſaw himſelf reduced to the neceſſity of either reſigning his poſt in the hoſpital, or of preſenting himſelf to the college. He determined to adopt the latter alternative, was examined and rejected. The cenſors with great candour had adviſed him to poſtpone his examination another year, if he thought himſelf not ſufficiently prepared; but he would not liſten to their advice. The expreſſion that had eſcaped Dr. Fothergill with regard to this unfortunate perſon, having come to his knowledge, he made it the foundation of an accuſation which was brought before his own ſociety. Theſe inoffenſive people who are averſe to the litigious proceedings that vex and ruin ſo many of their fellow citizens, referred the charge after their uſual manner, to a certain number of arbitrators. Five perſons were appointed for this purpoſe, and three of the number awarded 500l. damages to Dr. Leeds, after refuſing to hear Dr. Fothergill's principal evidence. The two other arbitrators with great propriety proteſted againſt the award; and after much altercation in the ſociety, Dr. Leeds moved the court of King's Bench to ſhew cauſe why the rule for the recovery of the damages ſhould not be made abſolute. Lord Mansfield, after hearing the evidence and counſel on the part of Dr. Leeds, refuſed to hear Dr. Fothergill's counſel; becauſe, he obſerved, the evievidence on the part of Dr. Leeds's arbitrators was ſufficient to prove the illegality and injuſtice of their own award. The learned and noble judge further added, that Dr. Fothergill did no more than his duty, in ſaying what he was charged with; and that he would not have acted as an honeſt man, had he ſaid leſs. This he illuſtrated by a facetious ſtory in which he had himſelf acted a ſimilar part with a diſcarded ſervant †. Another affair which occaſioned no little uneaſineſs to Dr. Fothergill, was a charge brought againſt him, by the editor of Parkinſon's voyage, of partiality, in adjuſting a difference between him and a reſpectable baronet. The doctor in a pamphlet entitled "Introductory re- "marks on the preface of Parkinſon's journal of "a voyage," very ably and ſatisfactorily vindicated himſelf from the aſperſions thrown upon him in this tranſaction.

So far was Dr. Fothergill from poſſeſſing a jealouſy at the appearance of a rival in phyſic amongſt thoſe of his own perſuaſion, that Dr. Chorley, a quaker phyſician, who had been educated under his old maſter, Benjamin Bartlett, was admitted into his own houſe; and at length, after being introduced into conſiderable practice, died under the roof of his patron. It might, indeed, truly be ſaid of Dr. Fothergill, that he was a liberal promoter of merit, without any regard to the religious tenets of its poſ-

† See Gent. Mag. for Nov. 1781.

feffor. It was in confequence of his recommendation that in 1780 Dr. Carmichael Smyth was fent to fuperintend the prifon at Winchefter, to avert, if poffible, the fpreading contagion of the gaol diftemper among the French and Spanifh prifoners. The fingular fuccefs of Dr. Smyth's attendance, whilft it did honour to his medical knowledge, reflected no lefs upon Dr. Fothergill's difcernment in the choice of an able phyfician. Long before this, we are told, he had been the means, and the fole means † of Dr. now Baron Dimfdale's being fent to St. Peterfburgh, where he defervedly acquired fo much honour and fortune.

Dr. Fothergill had very early acquired a tafte for botany, which he acknowledged to have been much heightened and improved by his friend Peter Collinfon. In proportion as the profits of his practice increafed, he indulged this tafte, and for this purpofe, in 1762, purchafed an eftate at Upton in Effex, containing about fixty acres of land, and between five and fix acres of garden ground. In this garden every plant that feemed likely to be of ufe in phyfic, or manufactures, was procured at any expence, and cultivated with the greateft attention. A winding canal, in the figure of a crefcent, nearly formed it into two divifions, and opened occafionally on the fight, through the branches of rare and exotic fhrubs on its banks. On this fpot, in the midft of winter, when the earth

† Dr. Lettfom's Life of Dr. F.

was covered with ſnow, evergreens were cloathed in full verdure. Without expoſure to the open air, a glaſs door from the manſion-houſe gave entrance into a ſuite of hot and greenhouſe apartments of nearly 260 feet extent, containing upwards of 3,400 diſtinct ſpecies of exotics. In the open ground, were to be ſeen about 3000 diſtinct ſpecies of plants and ſhrubs. From America he received various ſpecies of Catalpas, Kalmias, Magnolias, firs, oaks, maples, and other valuable productions, which became denizens of his domain, ſome of them capable of being applied to the moſt uſeful purpoſes of timber; and, in return, he tranſplanted green and bohea teas from his garden at Upton, to the ſouthern part of that great continent, now riſing into an independent empire. His ſupplies were not derived from America alone, for he ſpared no coſt in eſtabliſhing a univerſal correſpondence with almoſt every part of the habitable globe; and his wiſhes happily ſucceeded in procuring an immenſe variety of plants and ſeeds from China, and the Eaſt and Weſt Indies, from Siberia and the Alps, the new diſcovered iſlands, &c. and not a few from Africa, that ſtupendous garden of vegetable beauty. He endeavoured to improve the growth and quality of coffee in the Weſt India iſlands; the Bamboo cane (*Arundo Bambos*) calculated for various domeſtic uſes, he procured from China, and purpoſed to tranſplant it to our iſlands ſituated within the Tropics. To him and Dr. Ruſſell we are indebted for the flouriſhing of genuine ſcammony in our

 ſoil,

ſoil, as if indigenous to it. He attempted to procure the tree which affords the Peruvian bark, and is ſaid * to have at length ſo far ſucceeded, as to have had one plant in his garden, where it died. He likewiſe offered premiums of one hundred pounds each to two captains of ſhips for a plant in vegetation of the true † Winter's bark.

But

* Dr. Lettſom's account of Dr. F.

† Sir Joſeph Banks, bart. the learned preſident of the Royal Society, gives the following account of Dr. Fothergill's garden, in a letter to Dr. Thompſon, inſerted by the latter in his life of the doctor :

" At an expence ſeldom undertaken by an individual, " and with an ardour that was viſible in the whole of " his conduct, he procured from all parts of the world " a great number of the rareſt plants, and protected " them in the ampleſt buildings which this or any other " country has ſeen.

" He liberally propoſed rewards to thoſe whoſe cir- " cumſtances and ſituations in life gave them opportu- " nities of bringing hither plants which might be or- " namental, and probably uſeful to this country, or " her colonies; and as liberally paid theſe rewards to " all that ſerved him. If the troubles of war had per- " mitted, we ſhould have had the cortex Winteranus, " &c. &c. introduced by his means into this country; " and alſo the bread-fruit, mangaſteen, &c. into the " Weſt India iſlands. For each of theſe and many others " he had fixed a proper premium. In conjunction with " the Earl of Tankerville, Dr. Pitcairn, and myſelf, " he ſent over a perſon to Africa, who is ſtill employed " upon the coaſt of that country, for the purpoſe of " collecting plants and ſpecimens.

" Thoſe whoſe gratitude for reſtored health prompted " them to do what was acceptable to their benefactor, " were always informed by him that preſents of rare " plants chiefly attracted his attention, and would be

" more

But the exertions of Dr. Fothergill were not confined to botany. As he ftudied moft departments of natural hiftory, and patronized its ingenious cultivators, he neceffarily became poffeffed of a valuable collection of its rare objects. Next to the Duchefs of Portland, he had the beft cabinet of fhells ‡ in the kingdom. His

"more acceptable to him than the moft generous fees. "How many unhappy men, enervated by the effects "of hot climates, where their connections had placed "them, found health at their return home at that "cheap purchafe!

"What an infinite number of plants he obtained by "thefe means, the large collection of drawings he left "behind will amply teftify; and that they were "equalled by nothing but royal munificence, at this "time largely beftowed on the Botanic gardens at "Kew. In my opinion, no other garden in Europe, "royal, or of a fubject, had nearly fo many fcarce and "valuable plants.

"That fcience might not fuffer a lofs when a plant "he had cultivated fhould die, he liberally paid the "beft artift the country afforded to draw the new ones "as they came to perfection; and fo numerous were "they at laft, that he found it neceffary to employ "more artifts than one, in order to keep pace with "their increafe. His garden was known all over "Europe, and foreigners of all ranks afked, when "they came hither, permiffion to fee it."

‡ Dr. Fothergill had a very accurate knowledge of conchology: Mr. Da Cofta was indebted to him for many of the notes in his hiftory of fhells, and in a copy of Lifter's Exercitatio Anatomica de Cochleis Terreftribus, which was fold with the reft of his library, after his death, was found the following manufcript note: "This book I have corrected, and have made additions "for a fecond impreffion."—This laft anecdote has efcaped the notice of his other biographers.

colleccollection of ores and minerals was diftinguifhed for the rarity, rather than the number of the fpecimens that compofed it. His cabinet of infects was extremely elegant, and it was from the Doctor's collection of corals that the ingenious Mr. Ellis, F. R. S. delineated his fyftem. As a further proof of the attention Dr. Fothergill beftowed on Natural Hiftory, we may obferve, that his collection of exact and highly finifhed drawings of plants and flowers did not amount to lefs than twelve hundred. Thefe elegant fpecimens, the value of which it is difficult to eftimate, were chiefly on vellum by Ehret, Taylor, Harris, and Mifs Ann Lee, and were lately purchafed by the Emprefs of Ruffia for 2,300 pounds *. The great botanical work by Millar, in which the Linnæan fyftem is illuftrated with fingular elegance, was begun and finifhed under the patronage and infpection of Dr. Fothergill, to whom it was with great propriety fubfcribed. But the dedication was afterwards, with no little difficulty in recalling the copies, cancelled at his exprefs folicitation ‡. Though he took pleafure in encouraging ingenuity, he difliked to be told of it. Indeed he was averfe to dedications in general, and confidered them as a fpecies of literary pageantry, more productive of envy to the patron, than of advantage to the author.

Among other perfons patronized by this eminent phyfician was Anthony Purver, originally an unlearned Quaker mechanic, who had been

* Dr. Lettfom's account of Dr. F. ‡ Ibid.

brought brought up a ſhoemaker, with no other education than a very ſlender and imperfect knowledge of his native tongue. Being of a ſerious turn of mind, he reſolved to examine the religious ſentiments he had imbibed in his youth, and in the courſe of his enquiries found himſelf much embarraſſed by the different explanations of the ſcriptures. This determined him, though late in life, to ſtudy the original languages. He began with Hebrew, and in a very moderate compaſs of time made himſelf maſter of that and other oriental languages, which are moſt uſeful to a critical knowledge of the ſcriptures. He afterwards learned Greek, and at laſt Latin. The fruits of thoſe ſtudies were a new and literal tranſlation of the Old and New Teſtament, with notes critical and explanatory, in 2 vols. folio, printed in the year 1765 at the ſole expence * of Dr. Fothergill §.

At the approach of the ſevere winter of 1767, Dr. Fothergill propoſed a ſcheme, and liberally contributed to raiſe a fund for enſuring its ſucceſs, to purchaſe fiſh at a wholeſale cheap price, and to diſpoſe of them at a ſmall loſs, till the whole ſubſcription ſhould be expended. The ſociety, who ſupported this ſcheme, which was continued to the year 1770, in the ſame manner purchaſed potatoes in Lancaſhire, and conveyed them by water to the metropolis. And to countenance this diet, the Doctor purchaſed from

* This, we have been aſſured, did not fall much ſhort of £2,000.

§ Dr. Elliot's life of Dr. F.

the the warehoufes, opened for the fale of thefe arti-cles, the provifions of his own table, once at leaft every week *.

It was he who firft † fuggefted the plan of bringing fifh by land carriage, in order to break a monopoly which had highly enhanced the price of frefh fifh in all the markets about London.

His obfervations on fubjects of police, could they be collected together, would conftitute, we are told, an ample and ufeful volume ‡. He is faid to have written nearly an hundred letters in the Gazetteer on the fubject of the new pave-ment, and he was inceffantly communicating ufeful hints for the improvement of this great city. But of the different plans which his zeal to promote the welfare of fociety led him to un-dertake, the inftitution of the Seminary at Ack-worth, in Yorkfhire, was one of the moft im-portant to the prefent as well as to future times. This inftitution, of which he was the original projector in the year 1778, and to which he was a liberal benefactor, both during his life-time and by his will, at prefent contains above three hundred poor children of the fociety of Quakers, who are here furnifhed with all the neceffary con-veniencies and comforts of life, properly cloathed, and educated in every branch of knowledge fuit-able for the ftation in which it is prefumed they may be placed §.

Of his kindnefs and bounty to individuals

* Dr. Lettfom's life of Dr. F. † Ibid. ‡ Ibid.
§ Dr. Hird's affectionate Tribute, &c.

many many well attested anecdotes are related ||, and more might be collected. It will be sufficient here to mention his generosity to his worthy, but unfortunate friend, the late Doctor Gowin Knight, who applied to him in a moment of pecuniary distress, and returned with a heart set at ease by the noble benefaction of—a thousand guineas ‡.

Dr. Fothergill's ability to perform such acts of munificence was the result of a popularity which has been seldom equalled, perhaps never exceeded by any physician, and which continued undiminished as long as his health and strength would allow him to attend on his patients. How much he acquired in any one year is not with certainty known, because the exact extent of his profits he never disclosed, but it is certain that during a long series of years his diligence was unabated. He made every moment important by a wonderful regularity in his manners and in all his concerns, and his domestics had acquired a similar punctuality; and thus by general order and system, not a moment seemed with him to be lost in delays, or in his movements from one object to another. His engagements, however, in the way of his profession took such possession of his hours, that while his garden was every day inviting him by new objects of improvement and gratification, he may be almost said to have been tantalized by its pleasures.

|| Dr. Hird's affectionate Tribute, &c.

‡ Dr. Thompson's and Dr. Lettsom's Accounts of Dr. F.

In the day-time he was either at home attending to the complaints of a long fucceffion of patients, or vifiting them abroad with the utmoft frugality of time; and late at evening fo much taken up in anfwering confulting letters from diftant parts, that he could only borrow a few hours in a week to furvey his garden.

As this pleafant recefs was too remote to be often vifited, fo it was too much within the fphere of action to be a refuge from care and importunity, which yet the exhaufted ftate of his ftrength and fpirits required. This he determined upon; and having found a defirable fituation, at Lea-Hall, near Middlewich in Chefhire, he procured a leafe of it from the proprietor, Sir John Leicefter. This place was the more agreeable to him, as it was diftant only 18 miles from Warrington, where two of his brothers* then refided. To this fecluded fpot he firft retired in the fummer of the year 1765, and continued to make his anniverfary retreat to it as long as he lived, commonly leaving London in the month of July, and returning thither again in the beginning of October, fo as to be abfent eight or nine weeks.

On his return to the metropolis, his friends ufed to obferve with pleafure how much he was improved in complexion, chearfulnefs, and health. This retreat foon became the more neceffary, as in 1767 he removed from his long known refi-

* Samuel and Jofeph Fothergill. The former was a very eminent preacher amongft the Quakers. They both died feveral years before the Doctor.

dence in White Hart Court, to a new built houſe, which he purchaſed, in Harpur-ſtreet. The commodiouſneſs of this new ſituation ſoon preſented him with a very extenſive ſcene of buſineſs at the Weſt end of the town in families of rank, and at the ſame time his former connexions and friendſhips called for his daily attendance in the city, by which means his circle of practice became greatly enlarged.

During his retirement at Lea-Hall he uſed to enjoy the company of a ſmall number of friends, ride every day on horſeback, and purſue his plan and enlarge his ideas of horticulture. From his garden at Upton, he ſent duplicates of plants to Lea-Hall, and had the pleaſure of obſerving the peculiar effects of a new air, ſoil, and climate on vegetation. In this retreat he took no fees, but uſed to devote one day in the week to attend at Middlewich, and preſcribe gratis to all who applied to him. Here likewiſe he prepared many of his medical and other papers for publication, and wrote letters to his numerous correſpondents.

With North America his correſpondence was extenſive; his name was dear to the inhabitants; his father had thrice traverſed that continent* in the ſervice of religion; and his brother Samuel had followed the example of their parent. Many families, from the fame of his medical ſkill, croſſed the Atlantic to place themſelves under his care.

* Dr. Lettſom's Life of Dr. F.

With refpect to the late unfortunate conteft with our colonies, Dr. Fothergill, from its commencement, ftrenuoufly advifed leniency to our Tranf-atlantic brethren. His fentiments on this fubject are fully explained in an anonymous pamphlet, intitled, "An Englifh Freeholder's "Addrefs," publifhed in the year 1779, and of which, we now learn, he was the writer.

Between him and Dr. Franklin a mutual friendfhip had early commenced and continued to the death of the former. The American philofopher in a letter to Dr. Lettfom, dated Paffy, March 17, 1783, and printed by the latter in his life of Dr. Fothergill, after paying a due compliment to the philanthropy of his deceafed friend, fays, "juft before I left England, he in "conjunction with Mr. ** and myfelf, laboured "hard to prevent the coming war; but our en-"deavours were fruitlefs." This paffage alludes to a tranfaction which took place, it feems, in 1774, when a conference was held between Dr. Fothergill, Dr. Franklin, and another common friend, whofe name is not mentioned, for the purpofe of removing all differences between America and the Mother Country. At this meeting Dr. Franklin produced his propofitions *, and gave up fuch parts as were objected to by Dr. Fothergill and his colleague. In this ftate a copy of them was taken and imparted to fome perfons in power; and the anfwer was, that the propofitions were fuch as appeared to demand

* Thefe and other papers on the fame fubject are publifhed by Dr. Lettfom in his account of Dr. Fothergill.

too too much. Several attempts were afterwards made to reconcile the ſubjects of contention; but as the 12th article, which related to the repeal of the Boſton-port bill and of the acts which altered the charters of the Maſſachuſets Bay, and extended the limits of Canada, was inſiſted on by Dr. Franklin, though many of the others were acceded to, the negociation was broken off, and in a ſhort time afterwards Dr. Franklin embarked for America. The reader of ſenſibility who reflects on the train of evils that followed, and conſiders the fatal carnage of 100,000 victims of war, drawn from the loom and rural tillage, and with it the fruitleſs expenditure of 100 millions of money, muſt unavoidably regret, that the humane and patriotic endeavours of Dr. Fothergill and his friends were thus unhappily fruſtrated. Seeing, however, the impending danger, he perſevered in the ſame line of conduct, and renewed his endeavours to ſtop the effuſion of blood. Previous to the general aſſembly of the county of York, in 1779, he addreſſed a letter to his friend the rev. Mr. Zouch, of Sandal, in that county, a clergyman and magiſtrate of diſtinguiſhed reputation. This letter, which is penned in a clear, free, and maſculine ſtyle, full of argument and energy, was read in a committee of the meeting, and deſervedly met with the warmeſt approbation. "There is one neceſſary "point," ſays the Doctor in one part of it, "which, I think, you ought in the firſt place to "ſtate moſt clearly—the general decay of the "county—and keep cloſe to your own; manu-"factures declining, commerce languiſhing, va-

"lue

"lue of all land decaying, all public improve-

"ments at a ſtand, bankruptcies numerous,

"taxes increaſing, multitudes diſtreſſed, and,

"was it not for the late favourable ſeaſons, uni-

"verſal poverty and wretchedneſs muſt have

"taken place. Pray, therefore, that peace may

"be reſtored between us and America, as the

"only means of ſaving your county from every

"ſpecies of calamity;—the war with that coun-

"try, and its conſequences, having been the

"general cauſes of theſe diſtreſſes.—I do not

"mean that theſe expreſſions ſhould be uſed;

"you will find much better: but if you do not

"lay the axe to the root, in vain do you attempt

"the branches." It was not on men, but on meaſures, that the good Doctor animadverted. He uniformly mentioned his ſovereign in the moſt reſpectful language. "Let not a ſingle

"reflection on the king," ſays he in another part of the ſame letter*, "or the miniſtry,

"eſcape you—I mean not to appear in your pe-

"tition. The acrimony that loaded the Ame-

"rican petitions, and diſgraced many of our

"own, has done unſpeakable miſchief. I beg,

"therefore, and intreat, that every degree of in-

"vective may be ſhunned. Produce your facts,

"and place them in the cleareſt light; but if

"you mean well to your country, and wiſh to

"ſee an example followed in other counties,

"ſhun every thing offenſive. As there is no

"great room for flattery, ſo neither give way

* The whole of this excellent epiſtle is preſerved by Dr. Lettſom in his life of Dr. Fothergill.

"to the reverſe temper;--if you do, poſterity may

"load your memories with deſerved reproach."

For a ſeries of years, indeed for the moſt part of his life, Dr. Fothergill had enjoyed good health, and time ſeemed ſlowly to diminiſh the vigour of his body, or weaken the exertion of his mind. But in November 1778 he was afflicted with a diſorder which he apprehended, though without foundation, to be an irregular gout *. It terminated in a ſuppreſſion of urine, from which he recovered in about three weeks, and for the ſpace of two years enjoyed his wonted degree of health. He retired as uſual in 1779 and 1780 to Lea-Hall, and the laſt time returned by Buxton, where he projected thoſe improvements which we have already had occaſion to mention in our Journal (Vol. I. p. 276 and 434). He likewiſe viſited Knareſborough, in Yorkſhire, after many years abſence, to pay—as he relates in a letter to Dr. Lettſom—the grateful tribute of a tear at the ſide of his father's grave.

The time was now approaching when he himſelf was to experience, that neither temperance nor medical ſkill could exempt him from the final lot of humanity. On the 12th Day of December 1780 he was again ſeized with a ſuppreſſion of urine, which, notwithſtanding every effort of the experienced phyſicians and ſurgeon †,

* See the 1ſt vol. of our Journal, p. 195. The paper, of which an abſtract is there given, was read by Dr. Fothergill to the Society of Phyſicians, in July 1780, a few days before his departure for Cheſhire.

† Dr. Watſon, Dr. Warren, Dr. Reynolds, and Mr. Pott.

who who attended him, put a period to his existence on the 26th day of the same month.

On dissection, the disease appeared to have been occasioned by a schirrous enlargement of the prostate, which compressed the neck of the bladder, so as to prevent the introduction of a catheter. His remains were, on the 5th of January 1781, deposited in the Quakers burial ground at Winchmore-hill, about twelve miles from town; upwards of seventy coaches and post-chaises, filled with friends, attended on this melancholy occasion.

Dying a bachelor he bequeathed the bulk of his fortune to a maiden sister, who resided with him for many years before his death. He likewise bequeathed handsome legacies to his other relations and friends. His library *, which consisted of an excellent collection of books in physic and natural history, particularly the latter, has been sold by auction; and the late Dr. Hunter (another munificent promoter of science, whose loss we are just now deploring!) purchased his collection of shells, corals, and other curious subjects of natural history, for fifteen hundred pounds †.

* A collection of about 2000 English heads, in four volumes in folio and six in quarto, which formed a part of this library, was sold to Mr. Thane. These portraits, which had been collected by that ingenious antiquary the late Mr. John Nickolls, F. R. and A. S. (and which furnished Mr. Ames with his valuable catalogue) came soon after his death into the hands of Dr. Fothergill, who purchased them for 80 guineas.

† Dr. Fothergill gave directions by his will, that his collection should be appraised at his death; and that Dr. Hunter should have the refusal of it at five hundred pounds under the valuation.

The person of Dr. Fothergill * was of a delicate rather than an extenuated make. His features were all character, and his eye had a peculiar brilliancy of expression †. In his dress he was remarkably neat, plain, and decent.—At his meals he was uncommonly abstemious; eating sparingly, and rarely exceeding two glasses of wine at dinner or supper. By this uniform and steady temperance, he preserved his mind vigorous and active, and his constitution equal to all his engagements.

All who enjoyed the pleasure of his acquaintance ‡ agree, that he was a man endowed with many virtues and extraordinary abilities. His religious principles, conduct and services, made him highly respectable in his own society; at the same time that the chastity and integrity of his life and manners were universally known. His understanding was comprehensive, quick, and lively, present to the most sudden occasions, and very rarely embarrassed ||. There was a charm in his conversation and address that conciliated the regard and confidence of all who employed him, and so discreet and uniform was his conduct, that he was not apt to forfeit the esteem he had once acquired.

* Dr. Hird's Affectionate Tribute, &c.

† During his life-time he was often requested, but as often refused, to sit for his portrait. After his death it was very happily painted from memory by Mr. G. Stuart, an ingenious pupil of the celebrated Mr. West. From this picture, which appeared in the exhibition of the Royal Academy in 1781, an engraving in mettzotinto has been published by Mr. Green.

‡ Dr. Thompson's life of Dr. F. || Ibid.

Where he professed a friendship he was faithful and sincere; neither adversity nor death could dissolve it.

As he made the art of healing his principal study, so he was truly zealous for its honour and improvement. He was remarkably attentive to the diet of his patients; and was commonly among the foremost to make trial of new medicines that came recommended by experience, or their known sensible qualities.

His treatise on the putrid sore throat is alone sufficient to entitle him to a distinguished rank amongst medical writers. His other productions, it must be confessed, are of inferior merit. His reputation will certainly not be enhanced with posterity by swelling the catalogue of his works, and the Medical Society, of which he was a member, have shewn a true regard to his memory by suppressing some of his manuscript essays, which had been hastily conceived, and were too incorrect to obtain his own approbation *. We wish to see this laudable mode of conduct more generally adopted; posthumous publications would then be more favourable than they are at present to the reputation of authors. The fame of many an admired writer has suffered through the mistaken zeal of his friends or the avarice of a bookseller.

As a citizen of the world, attentive to the wants and miseries of mankind, and constantly busied in promoting schemes of public utility, and the improvement of arts and sciences, Dr.

* Dr. Thompson's life of Dr. F.

Fother-

Fothergill's character will suffer no diminution compared with any person of the present age.

Where so many good qualities resided it might appear invidious to point out trifling foibles. Some such fell to the share of Dr. Fothergill. They were, however, more than compensated by his virtues; and the public, as well as his friends, hath sustained, by his death, an irreparable loss.

