# Errata

**Published:** 1783

**Authors:** 


					p. 178, /. J 6, for Re Emetico-
rum ufu read De Emeticorum ufu $

				

